# Unveiling the Frailty Spatial Patterns Among Chilean Older Persons by Exploring Sociodemographic and Urbanistic Influences Based on Geographic Information Systems: Cross-Sectional Study

**DOI:** 10.2196/64254

**Published:** 2025-04-17

**Authors:** Yony Ormazábal, Diego Arauna, Juan Carlos Cantillana, Iván Palomo, Eduardo Fuentes, Carlos Mena

**Affiliations:** 1 Longevity Center VITALIS, Faculty of Economics and Business, University of Talca, Universidad de Talca, Avenida Lircay S/N, Talca, 3460000, Chile, 56 712200200; 2 Thrombosis Research Center, Department of Clinical Biochemistry and Immunohematology, Faculty of Health Sciences, Interuniversity Center of Healthy Aging (CIES), Longevity Center VITALIS, University of Talca, Talca, Chile; 3 Facultad de Administración y Economía, Universidad Tecnológica Metropolitana, Santiago, Chile

**Keywords:** aging, frailty, geospatial clustering, urban factors, neighborhood conditions.

## Abstract

**Background:**

Frailty syndrome increases the vulnerability of older adults. The growing proportion of older adults highlights the need to better understand the factors contributing to the prevalence of frailty. Current evidence suggests that geomatic tools integrating geolocation can provide valuable information for implementing preventive measures by enhancing the urban physical environment.

**Objective:**

The aim of this study was to analyze the relationship between various elements of the urban physical environment and the level of frailty syndrome in older Chilean people.

**Methods:**

A cohort of 251 adults aged 65 years or older from Talca City, Chile, underwent comprehensive medical assessments and were geographically mapped within a Geographic Information Systems database. Frailty was determined using the Fried frailty criteria. The spatial analysis of the frailty was conducted in conjunction with layers depicting urban physical facilities within the city, including vegetables and fruit shops, senior centers or communities, pharmacies, emergency health centers, main squares and parks, family or community health centers, and sports facilities such as stadiums.

**Results:**

The studied cohort was composed of 187 women and 64 men, with no significant differences in age and BMI between genders. Frailty prevalence varied significantly across clusters, with Cluster 3 showing the highest prevalence (14/47, *P*=.01). Frail individuals resided significantly closer to emergency health centers (960 [SE 904] m vs 1352 [SE 936] m, *P*=.04), main squares/parks (1550 [SE 130] m vs. 2048 [SE 105] m, *P*=.03), and sports fields (3040 [SE 236] m vs 4457 [SE 322]m, *P*=.04) compared with nonfrail individuals. There were no significant differences in urban quality index across frailty groups, but frail individuals lived in areas with higher population density (0.013 [SE 0.001] vs 0.01 [SE 0.0007], *P*=.03).

**Conclusions:**

Frail individuals exhibit geospatial patterns suggesting intentional proximity to health facilities, sports venues, and urban facilities, revealing associations with adaptive responses to frailty and socioeconomic factors. This highlights the crucial intersection of urban environments and frailty, which is important for geriatric medicine and public health initiatives.

## Introduction

Understanding the aging process and the sociodemographic determinants related to enhancing the quality of life has emerged as a very relevant research area in light of the rapid aging of the global population [1-3]. Currently, 12% of the world’s population is aged ≥60 years, and projections suggest that this proportion may rise to 21.5% by the mid century [4]. Similarly, the ≥80 years age group is expected to increase from 1.7% to 4.5% [4]. In this context, Chile is experiencing a pronounced aging phenomenon [4,5]. Projections indicate that the Chilean population aged ≥60 years is set to surge from 15.7% to 32.9% by 2050, with the proportion of individuals aged ≥80 years potentially reaching 10.3% [4].

According to the World Health Organization, the frailty syndrome is a crucial determinant regarding the state of dependency, the presence of chronic diseases, polypharmacy, and the quality of life in older people [6,7]. The frailty syndrome is defined as a preventable and reversible clinical state in which the capacity of older people to cope with everyday stressors is compromised by an increase in vulnerability and the physiological deterioration associated with aging [8]. Recent results show a prevalence of frailty in Chile slightly higher than 20% [9,10]. Frail persons have higher risks of mortality, cognitive impairment, fractures, and hospitalization, among other adverse health events, which, considering the increase in the population of older people, represents a challenge for public health and social welfare systems [11,12].

The built environment refers to spaces altered or created by human activities, encompassing a spectrum from homes and schools to workplaces, highways, urban expanses, accessibility to amenities, recreational areas, and pollution [13]. This environment can be delineated into 2 primary components: the microenvironment, encapsulating neighborhood and street-level attributes, and the macroenvironment, which includes the degree of urbanization and patterns of land use [14]. Enhancing our understanding of how the urban physical environment impacts older adults can significantly aid in formulating effective plans and interventions to prevent the progression and onset of frailty while promoting the well-being of this population [15]. Previous research conducted by our group has demonstrated that leveraging geomatic tools, which integrate geolocation as an additional dimension of analysis, can provide valuable insights for studying frailty as a syndrome and supporting the implementation of preventive measures [16,17].

According to reports from the World Health Organization on aging and friendly cities, enhancing the environment through improvements in physical structures and community support is an effective approach to maintaining the health of older people [18]. Recent evidence underscores the impact of neighborhood characteristics on frailty among older people. Those residing in neighborhoods with abundant green spaces exhibit a lower incidence of frailty, whereas individuals perceiving precarious conditions in their surroundings, houses, and environment face a higher risk of frailty [19-21]. A comprehensive multilevel (individual and community) cross-sectional analysis highlighted that older adults living in aesthetically pleasing and walkable neighborhoods tend to exhibit lower levels of frailty. In contrast, areas with high-traffic roads, for example, were associated with a higher prevalence of frailty [22,23]. These findings emphasize the critical role of physical environmental factors in shaping the health and well-being of older populations, highlighting the importance of designing age-friendly communities that promote active and healthy aging.

In this context, this study aims to analyze the relationship between various elements of the urban physical environment and the level of frailty syndrome in older Chilean people.

## Methods

### Participants and Study Design

The research adopted a cross-sectional case-control design, with a representative sample of older persons (aged ≥65 years old, both men and women) randomly selected from various Family Health Centers and community groups of older people in Talca City, Chile (n=251). All medical centers that serve older adults in the city were considered, ensuring geographical representation. The inclusion criterion was adults aged 65 years and older. Participants with self-reported or medically documented cancer, Parkinson disease, or vascular events were excluded, as were older individuals unable to walk or speak [9]. The calculation of the sample size (aged ≥65 years old, both men and women) considered a prevalence of frailty in older adults of 24.6% [9], with a 95% CI, statistical power of 80%, and a loss percentage of 20%. The proportions of women and men in the sample were determined by the relative distribution of the adult population over 65 years using data from the National Socioeconomic Characterization Survey [24]. No additional stratification was applied.

### Frailty Diagnosis

The Fried frailty phenotype criteria were used as the diagnostic tool for assessing frailty [9,25]. This method evaluates the presence or absence of the following components: slowness, weakness, weight loss, exhaustion, and low physical activity. These parameters were defined based on the criteria described previously by Palomo et al [9], which include: (1) slowness: walking velocity below a cut-off of 0.8 m/s average 3-meter walk at a usual pace, adjusted for sex and height according to the standards of the Short Physical Performance Battery, (2) weakness: handgrip strength measured using an Electronic Handgrip Dynamometer (Camry), with sex-specific cut-off (male <27 kg, female <15 kg), (3) weight loss: defined as loss of at least 5 kg in the previous 6 months, (4) exhaustion: a positive response to any of the following two questions from the Center for Epidemiological Studies Depression Scale: “I felt that anything I did was a big effort” and “I felt that I could not keep on doing things” at least 3 to 4 days a week,” (5) low physical activity: difficulty walking, assessed by the questions “Do you have difficulty walking a block?” or "Do you have difficulty climbing several flights of stairs without resting? Participants meeting 3 or more of these components were categorized as frail, those with no 1 or 2 components were considered prefrail, and individuals lacking all components were classified as non-frail or robust [9].

### Geospatial Clusters

**Figure 1.**
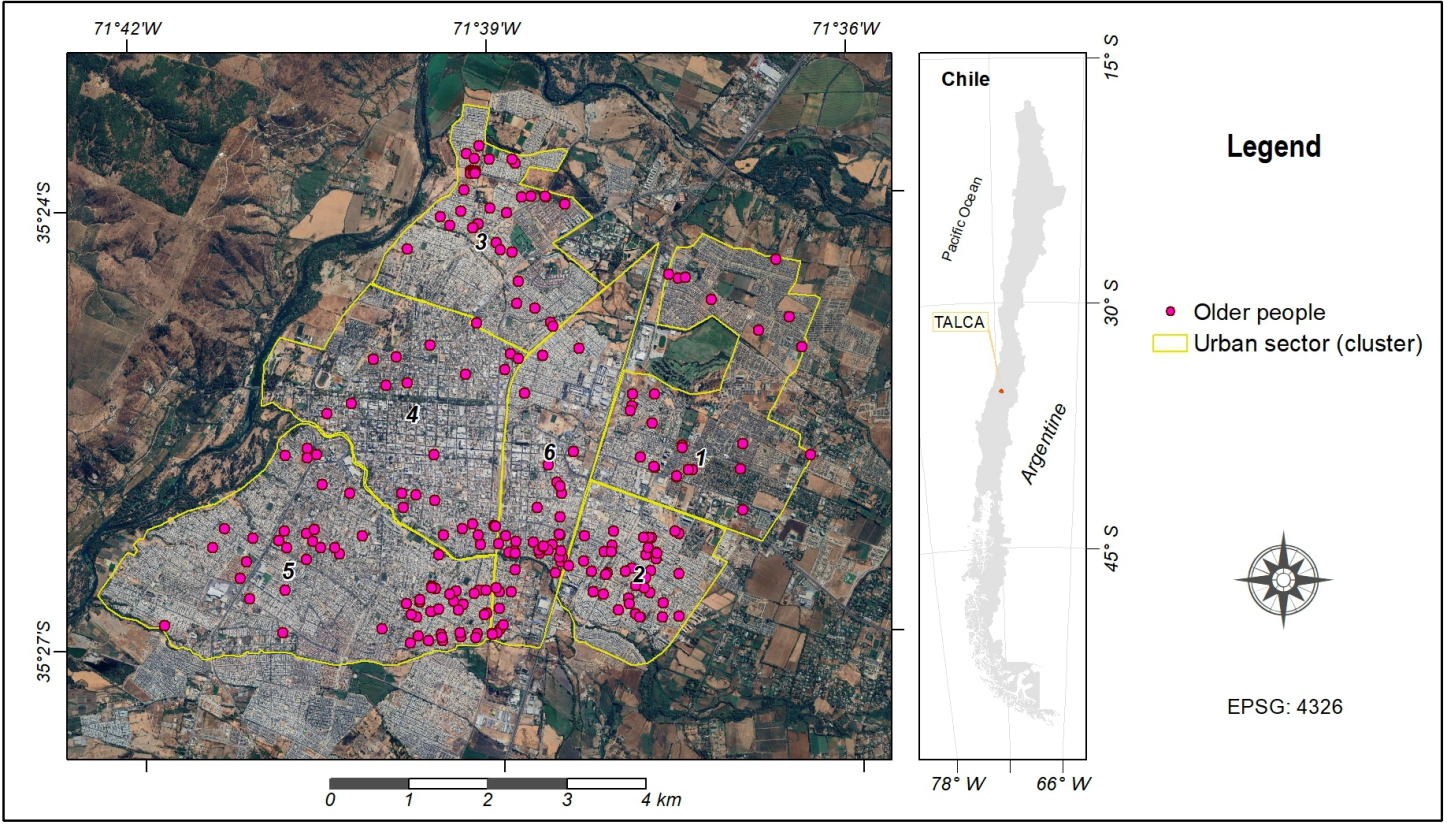
Location of older individuals within the 6 urban sectors.

Each participant was geographically located according to the city address informed and represented as a point object based on the residence information provided during the medical evaluation. All data were organized into a point feature layer accompanied by its corresponding thematic table. This layer was integrated into a geodatabase for subsequent analysis within its geographical context, along with pertinent factors related to the urban physical environment. Figure 1 displays the individual residency positions of each older adult participating in the study within their respective geographical cluster. These geographical areas were delimited in the city of Talca, Maule Region, Chile, based on geospatial location and sociodemographic characteristics. Cluster 1 corresponds to the northeastern sector characterized by high socioeconomic status. Cluster 2 encompasses the southeastern sector with low socioeconomic status. Cluster 3 covers the northern sector with a lower-middle socioeconomic class, and Cluster 4 represents the “historic center” area. The southern sector of medium-high socioeconomic level is covered by Cluster 5, and Cluster 6 corresponds to the “industrial center” area.

### Urban Quality Level

**Figure 2.**
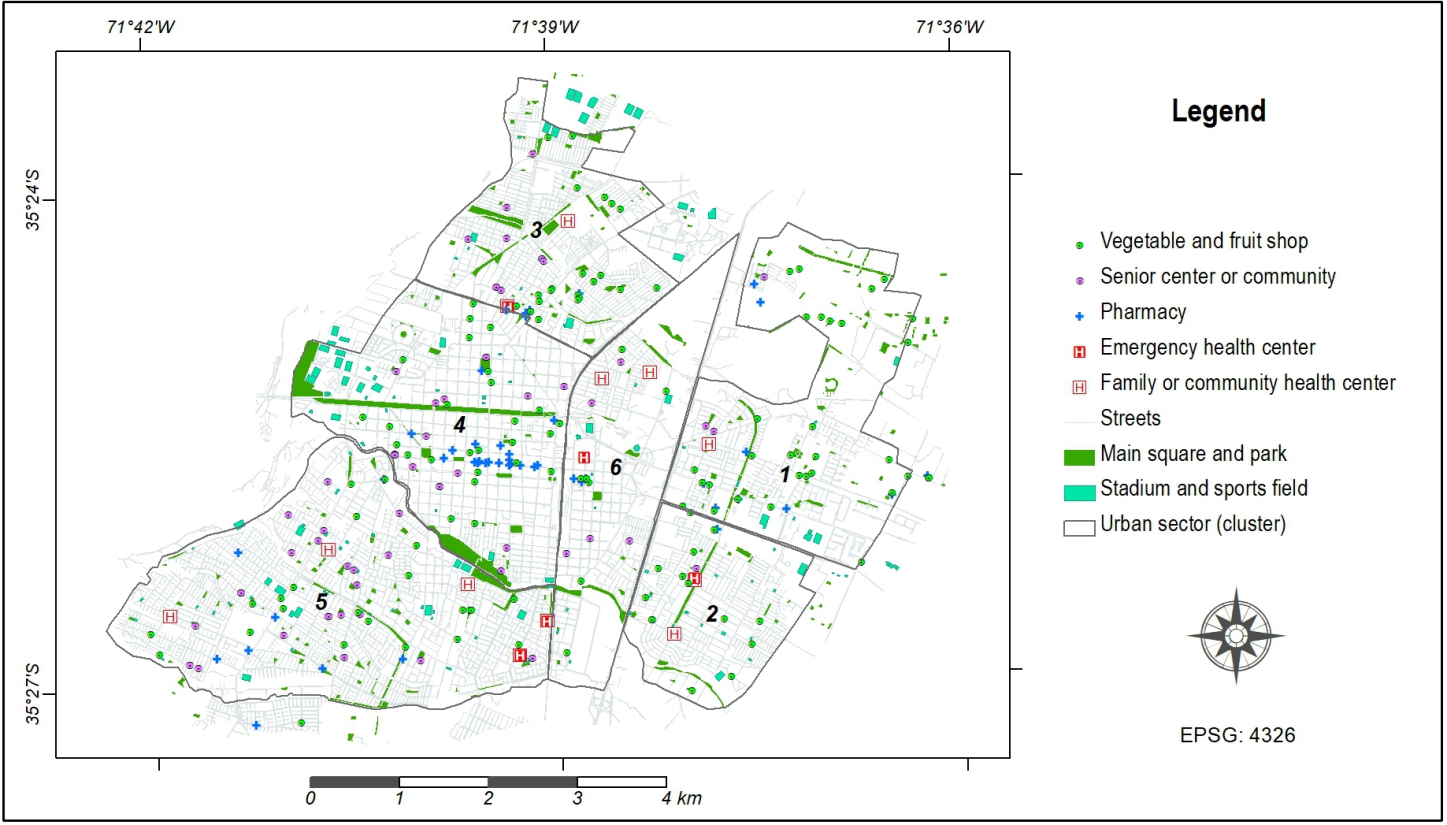
Geographical distribution of urban physical facilities within Talca City.

A georeferenced database was constructed using Geographic Information Systems (GIS) technology to represent pertinent geographical information concerning urban physical facilities within the city, encompassing: (1) vegetables and fruits shops, (2) senior centers or communities, (3) pharmacies, (4) emergency health centers, (5) main squares and parks, (6) family or community health centers, and (7) stadiums and sports fields. Each component within the study area was depicted as a GIS layer, either in point or polygon form, at the neighborhood scale. This representation (Figure 2) was derived from data sourced from OpenStreetMap [26], Google Maps [27] and Infraestructura de Datos Geospaciales de Chile (IDE Chile) [28]. Subsequently, each GIS layer underwent analysis using the Euclidean distance method, providing insights into the proximity of every location within the city to the considered infrastructure. The resulting distance layers were subsequently classified to delineate 3 distinct zones encircling the urban facilities, categorizing their proximity as either close, medium, or distant. Next, each proximity class for every layer was assessed on a scale ranging from 1 to 3, wherein the closest proximity received a score of 3, and the more distant areas were assigned a score of 1. The distance ranges, and the corresponding values assigned to each urban facility were defined according to local context and are presented in Table 1. A general criterion for evaluation was that the closer the facility, the higher the value assigned. A raster calculator was used to aggregate all layers, yielding a summary index where a higher numerical value signifies enhanced urban quality in the depicted area. Afterward, the values derived from the distance analyses and the corresponding summary index for each participant were integrated into the point feature layer. Management, processing, and analyzing data were performed using ArcGIS software, version 10 (ESRI, Redlands, USA).

**Table 1.** Distance ranges and values for each urban physical facility.

Facility and distance ranges	Value
**Vegetables and fruit shops (m)**	
<300	3
300‐600	2
>600	1
**Senior centers or communities (m)**	
<500	3
500‐1000	2
>1000	1
**Pharmacies (m)**	
<500	3
500‐1000	2
>1000	1
**Emergency health centers (m)**	
<1000	3
1000‐2000	2
>2000	1
**Main squares and parks (m)**	
<200	3
200‐400	2
>400	1
**Family or community health centers (m)**	
<700	3
700‐1400	2
>1400	1
**Stadiums and sports fields (m)**	
<400	3
400‐600	2
>600	1

### Statistical Analysis

Statistical analyses were conducted using GraphPad Prism 9. Continuous variables were expressed as mean (SD) or median (95% CI). Categorical variables were expressed as percentages with a 95% CI. In the evaluation of differences between groups, the chi-square test with Yate correction was used to assess proportions, while ANOVA or the Kruskall-Wallis test, as appropriate, was applied to assess differences in means or medians. Statistical significance was considered at *P* values below .05.

### Ethical Considerations

The institutional board review approval for this study was obtained from the Comité de Ética Científica (CEC) of Universidad de Talca (reference number 06‐2021). All procedures followed adhered to the ethical standards of the CEC and the World Medical Association’s Declaration of Helsinki. All study participants provided written informed consent.

## Results

### Sociodemographic Characteristics and Cluster Distribution

Table 2 presents the sociodemographic characteristics of the analyzed cohort of older people. The sample comprised 74.5% women and 25.2% men, with no significant differences observed in age and BMI between the 2 genders. In addition, Table 2 illustrates the distribution of the geospatial clusters established during the study. Analysis indicated no significant difference in the distribution of the 6 designated clusters between men and women.

**Table 2.** Sociodemographic description and geospatial distribution of the studied sample of older people.

Variable	Women (n=187)	Men (n=64)	*P* value
Gender, % (95% CI)	74.5 (68.7-79.4)	25.5 (20.5-31.2)	—^a^
Age (years), mean (SD)	73.8 (5.9)	75 (5)	.152
BMI (kg/m^2^), mean (SD)	33.6 (33.5)	28.4 (6.5)	.243
**Spatial cluster showing percentage of prevalence, % (95% CI)**			
Cluster 1	10.2 (6.6-15.3)	15.6 (8.7-26.4)	.260
Cluster 2	18.7 (13.7-24.9)	14.1 (7.6-24.6)	.452
Cluster 3	16.6 (11.9-22.5)	25 (16-36.8)	.142
Cluster 4	11.2 (7.5-16.6)	9.4 (4.4-18.9)	.817
Cluster 5	26.2 (20.4-32.9)	17.2 (9.8-28.2)	.176
Cluster 6	17.1 (12.4-23.2)	18.8 (11.1-29.9)	.845

a —: not applicable.

### Analysis of Urban Quality

**Figure 3.**
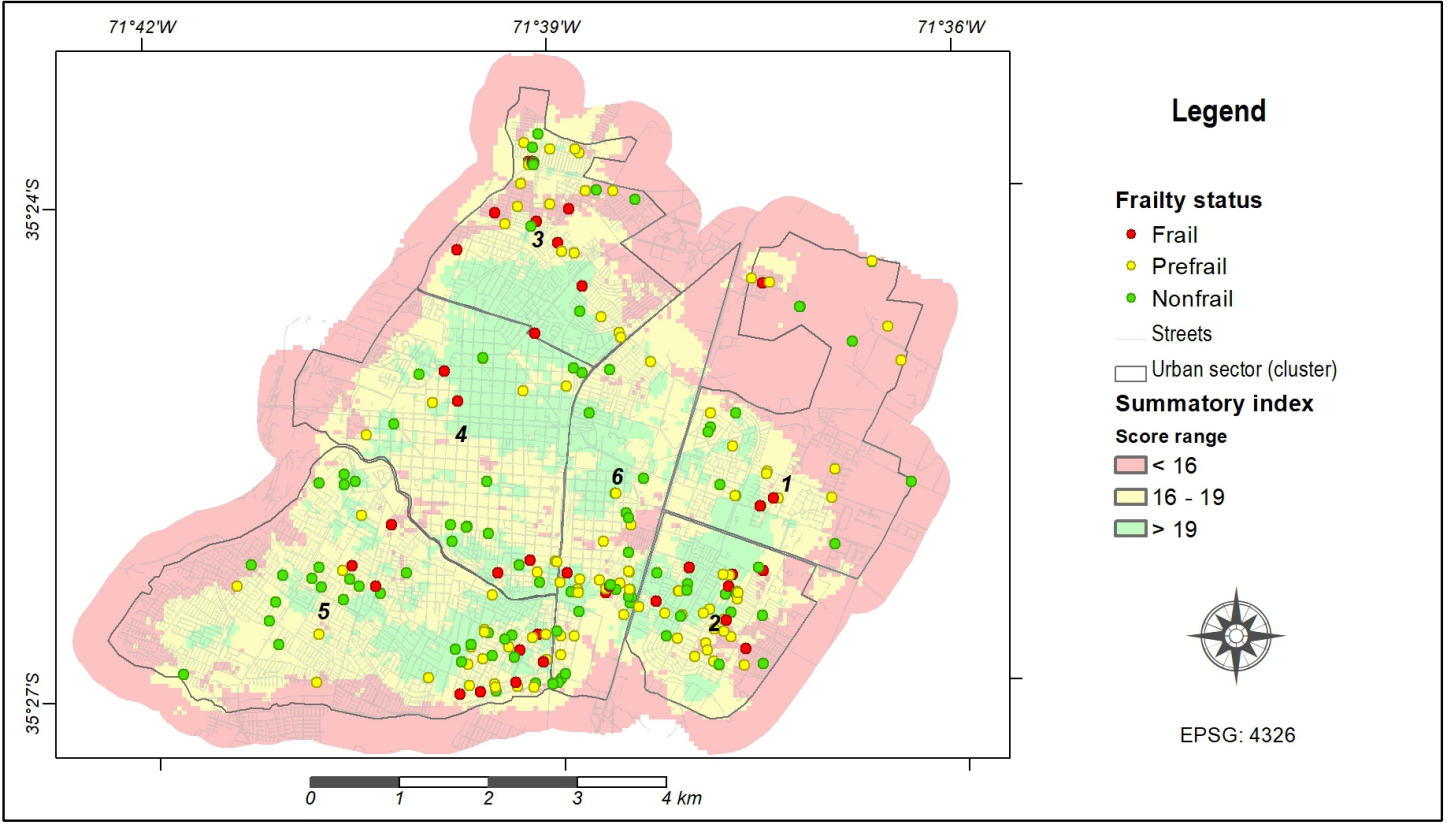
Frailty status of older individuals over summary index within the geospatial clusters defined for Talca city.

Figure 3 shows the different levels of urban quality [3] obtained from the cumulative assessment of physical environmental elements considered in this study. These elements mainly encompass basic urban services and infrastructures essential for the local population, with particular importance for the cohort of older people under study. The quality level is closely related to the accessibility of the various facilities from each location within the city. In addition, Figure 3 also shows the individual distribution of older adults, each denoted by their frailty status, which is subsequently analyzed in Table 3. This table displays the distribution of the frailty status through the different geospatial clusters. The prevalence of frailty varies between 7.3% and 34.1% among these clusters. Notably, cluster 3 exhibits a significantly high prevalence of frail people (34.1%, *P*=.006), followed by cluster 5 (21.9%, *P*=.417); however, this last prevalence is not significantly high.

**Table 3.** Distribution of older people by frailty status and geospatial clustering.

Spatial cluster	Frailty status considering the percentage of prevalence			*P* value
	Nonfrail (%, 95% CI)	Prefrail (%, 95% CI)	Frail (%, 95% CI)	
Cluster 1	14.3 (8.5‐22.9)	11.7 (6.9‐19)	7.3 (2.5‐19.4)	.620
Cluster 2	14.3 (8.5‐22.9)	21.6 (14.9‐30.2)	17.7 (8.5‐31.3)	.270
Cluster 3	12.1 (6.8‐20.4)	19.8 (13.5‐28.2)	34.1 (21.5‐49.5)	.006
Cluster 4	14.3 (8.5‐22.9)	8.1 (4.3‐14.6)	12.2 (5.3‐25.5)	.477
Cluster 5	30.7 (22.2‐40.9)	20.7 (14.2‐29.2)	21.9 (12‐36.7)	.417
Cluster 6	14.3 (8.5‐22.9)	18 (11.9‐26.2)	7.3 (2.5‐19.4)	.225
Total	100	100	100	—^a^

a —: not applicable.

### Analysis of Distances to Urban Facilities, Summary Index, and Population Density

**Figure 4.**
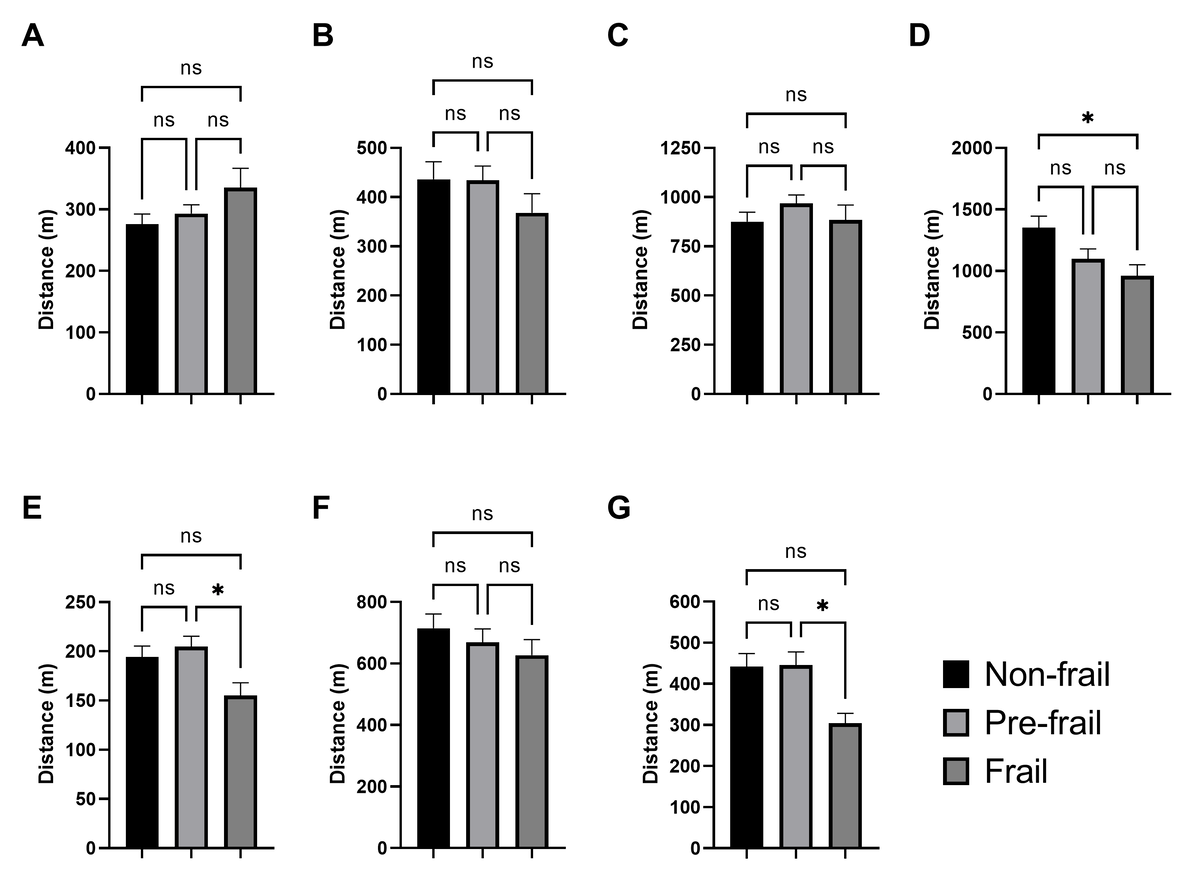
Comparison of the mean distance for different frailty status diagnosed using the Fried phenotype criteria with respect to relevant urban facilities of (A) vegetables and fruits shops; (B) senior centers or communities; (C) pharmacies; (D) emergency health centers; (E) main squares and parks; (F) family or community health centers; (G) stadiums and sports fields. The data presented are the mean (SE). Statistical analysis was performed using the ANOVA test with the Tukey pos-hoc test. *P<.05.

**Figure 5.**
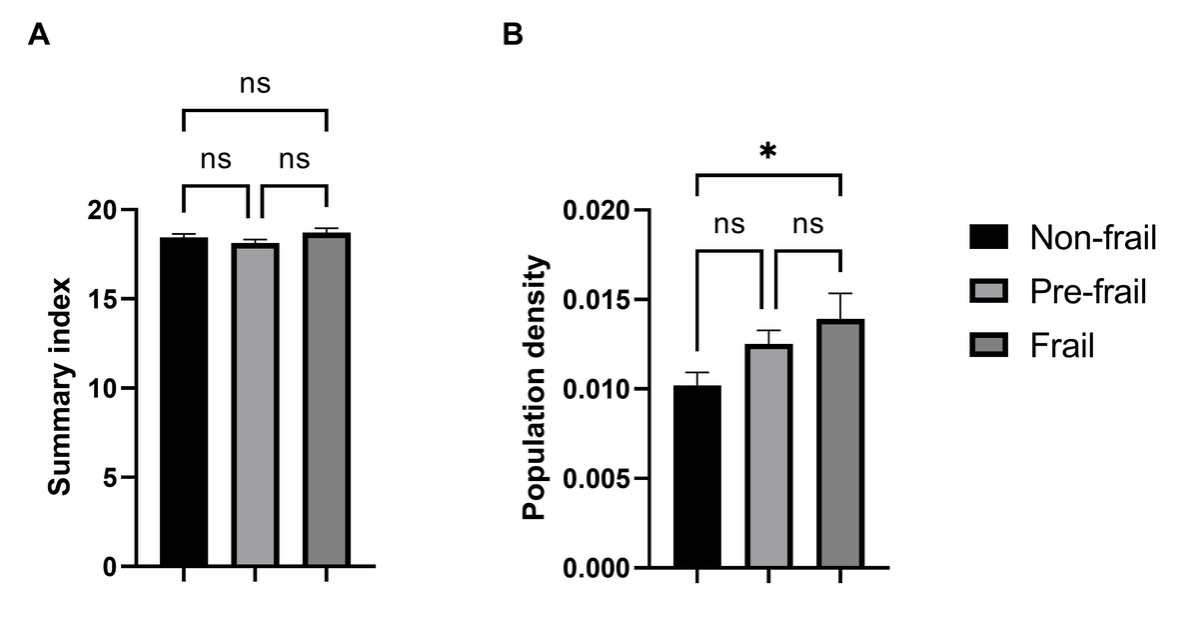
Comparison of mean values for different frailty status diagnosed according to the Fried phenotype criteria for the (A) summary index, (B) population density. The data presented are mean (SE). Statistical analysis was performed using the ANOVA test with the Tukey post-hoc test. *P<.05.

Figure 4 illustrates the variation in average distance between old persons (categorized by their frailty status) and relevant urban facilities. For the facilities of vegetable and fruit shops (A), senior centers or communities (B), pharmacies (C), and family and community health centers (F), there were no significant differences in the average distance across different frailty status groups. However, a clear linear trend is observed between the groups, where frail people tend to reside further from vegetable and fruit shops (frail: 335.6 [SE 31.2] vs nonfrail: 275.9 [SE 16.5]) and closer to the senior centers or communities (frail: 368.2 [SE 38.6] vs nonfrail: 435.9 [SE 35.9]) than the nonfrail people. On the other hand, the facilities of emergency health centers (D), main squares and parks (E), and stadiums and sports fields (G) present significant differences in the average distance across different frailty status groups, where frail people resided significantly closer to emergency health centers (frail: 960.4 [SE 90.4] vs nonfrail: 1352 [SE 93.6], *P*=.04), main squares and parks (frail: 155 [SE 13] vs prefrail: 204.8 [SE 10.5], *P*=.03), and stadiums and sports fields (frail: 304 [SE 23.6] vs prefrail: 445.7 [SE 32.2], *P*=.04), than both nonfrail and prefrail people, respectively. Finally, Figure 5 shows the variations in the summary index and population density between the frailty status groups. While the summary index (A) shows no significant differences between groups, there is a significant difference in population density (B) between the frail and nonfrail status groups, being higher for frail people (frail: 0.013 [SE 0.001] vs nonfrail: 0.01 [SE 0.0007], *P*=.03).

## Discussion

### Principal Findings

This study explores the relationship between urban physical environment factors and frailty syndrome, with a particular focus on its diagnostic criteria. The results mentioned above indicate that frail individuals tend to live closer to emergency health centers, stadiums, sports fields, and senior centers, suggesting efforts to improve their health and social engagement. In addition, frail individuals primarily reside in densely populated areas, which are associated with limited physical activity and higher mortality rates. Given the relevance of the Fried frailty phenotype as a standard frailty assessment tool in various studies involving older adults, it was selected for frailty characterization [11,29,30]. Investigating the link between frailty and urban environment is innovative and can provide invaluable insights for government programs aimed at enhancing the well-being of older people [31,32]. However, this emerging topic remains underdeveloped, limiting opportunities for comparisons specific to frailty. The incorporation of geomatics tools enables the use of geolocation as an additional analytical dimension, offering valuable insights into the study of frailty as a syndrome. This is especially important when the primary focus is on understanding the impact of the urban physical environment on the frail condition of older individuals [16,17]. Currently, the use of information and communication technologies among older persons is increasing, and different benefits have been developed about frailty with respect to predicting the risk, assisting in identifying changes in frailty parameters, enhancing adherence to a healthy diet, and distinguishing older rehabilitation patients who need to be readmitted to the hospital from those who can remain in the community which could facilitate this key process [33-37].

The data from Table 2 indicate homogeneity within the cohort, with no significant influences in the study parameters when stratified by gender. Participants had an average age range of 73‐75 years old and exhibited a high BMI. This trend of elevated BMI is consistent with findings from other studies focused on older Chilean people, wherein a significant prevalence of obesity (BMI >25 kg/m^2^) has been documented [8,9,38]. Gender did not appear to influence the distribution prevalence within the identified clusters. However, it is necessary to account for gender in geospatial clustering analyses involving older people, given its potential impact on various health outcomes, as recently evidenced in COVID-19 studies [39].

The findings presented in Table 3 suggest that geospatial distribution significantly impacts the prevalence of frailty syndrome. Notably, clusters 3 and 5, which are associated with middle to low socioeconomic classes, concentrate more than half of the frail people. This evidence is consistent with studies where low socioeconomic groups have been associated with a higher risk of developing frailty [31]. Furthermore, previous research indicates that frailty condition among older people tends to exhibit spatial clustering, wherein certain areas within and outside the city display localized concentrations of both high and low prevalence of frail people [16,40]. These clustering patterns can be attributed to the diverse urban infrastructures and socioeconomic disparities observed across different sectors within the city [16].

In this study, the outcomes depicted in Figure 3 reveal a geospatial clustering concentrated in the southern part of the city, primarily associated with clusters 3 and 5. However, when considering the general condition of the urban physical environment, characterized by the summary index, the group of frail people does not show significant differences compared with the other groups (Figure 5A). This fact suggests that, despite various groups of adults residing in environments characterized by comparable habitability conditions, distinctions in certain factors, as evidenced in Figure 4, can exert relevant positive or negative influences. Thus, the urban physical environment is likely connected with concurrent factors associated with the frailty syndrome, serving either as causal factors or as responses to such influences.

Frailty is acknowledged as a critical factor influencing the health and well-being of older people [11-18]. It stands out as the primary risk factor and indicator for the initiation of dependency, as well as the occurrence of chronic diseases, hospitalizations, falls, fractures, and mortality. The extensive array of health-related challenges observed in frail people may be associated with their proximity to emergency health centers, as well as family or community health centers [15-20]. Likewise, frailty has exhibited strong associations with obesity, sarcopenia, and low physical activity. Consequently, the proximity identified between the frail group and stadiums and sports fields may suggest a purposeful endeavor to improve body composition and address underlying clinical conditions [41,42].

Simultaneously, the frail group exhibited greater proximity to main squares and parks and to senior centers or communities renowned for fostering social activities among older people [43]. Given the well-established associations between frailty and social isolation, depression, and loneliness [44], the observed significant trend might signify a proactive response to enhance community engagement. This trend could also be indicative of the city government’s concerted efforts to provide these facilities to a population in need. By strategically situating frail individuals near these spaces, urban planners and policymakers may be aiming to reduce the adverse effects of isolation and encourage greater social participation. Engaging in recreational activities and interacting with peers in accessible public spaces can help mitigate the mental health challenges often faced by frail older adults. Furthermore, these spaces serve as venues for both physical and mental stimulation, which are critical to maintaining functional independence and improving overall well-being.

Conversely, the robust association between frailty and obesity might elucidate the trend observed trend of increased distance from the frail group to fruit and vegetable shops. Nevertheless, validation of this hypothesis necessitates examination within a more extensive cohort. While elevated intake of fruits and vegetables has been linked to a diminished frailty risk, the impact of the proximity of these food supply points on frailty remains ambiguous [45]. Furthermore, greater distances to pharmacies may indicate challenges in accessing essential drugs and medications, a factor that should be taken into account in health programs tailored to support the frailty group.

The findings depicted in Figure 5B indicate that the frail group tends to inhabit regions characterized by elevated population density. Older adults tend to live in densely populated marginalized areas due to economic, social, and structural factors. Financial limitations, the lack of adequate housing options, access to family support networks, and mobility barriers are some of the key factors influencing their stay in these areas. This circumstance poses an increased risk to this group, as current scientific understanding suggests a positive correlation between population density in a given neighborhood and increased susceptibility of middle-aged and older adults to overweight conditions [46]. This association can be ascribed to the proclivity of individuals residing in densely populated areas to adopt a sedentary lifestyle, marked by limited physical activity and diminished energy expenditure [20,46]. Notably, low physical activity and sedentarism are integral components of the frailty phenotype observed across diverse cohorts of older adults [11,12,26]. Furthermore, elevated population density has been lined to increased mortality rates across all causes in older people [11,12,47,48].

Initiatives aimed at preventing frailty underscore the imperative to advocate for physical activity, nutrition, and social engagement as primary and efficacious interventions. These interventions can be effectively implemented through a health education program tailored to inspire and engage older people [49,50]. Our findings underscore the importance of taking urban factors into account when examining the frailty condition of older people. This evidence aligns with previous research emphasizing the importance of these factors in the well-being and social engagement of older adults, as well as their association with frailty [16,51]. These results emphasize the imperative to investigate further and enhance our comprehension of the role played by urban factors in shaping frailty among older people. Regardless, it is imperative to consider the complex interrelationship between the urban physical environment and frailty condition when devising structural preventive measures aimed at improving the well-being of older people [16,47].

### Conclusions

Contemporary evidence underscores the relevance of urban factors in influencing the onset of frailty and the diverse health factors linked to this syndrome. Frailty stands as a highly prevalent geriatric syndrome in the elderly, elevating the susceptibility to a range of adverse health and social outcomes. Addressing this challenge necessitates the development of age-friendly cities tailored to the needs of older populations. Our findings suggest that individuals classified as frail tend to reside in closer proximity to emergency health centers, as well as family or community health centers, which may be indicative of adaptive responses to the features associated with frailty, such as elevated mortality risk and diminished levels of physical activity. Likewise, frail people tend to reside closer to stadiums and sports fields, which may imply a deliberate endeavor to enhance body composition and address underlying clinical conditions. On its part, the closer proximity of frail people to urban infrastructures such as main squares and parks, and senior centers or communities may be indicative of the concerted effort by the municipal government to provide these facilities to a population in need. However, it is important to highlight that the frail group predominantly inhabits areas characterized by elevated population density, aligning with earlier research associating higher mortality rates with increased population density among older adults. Likewise, the geospatial clustering highlights the relevance of both socioeconomic status and geographic location in relation to frailty prevalence, unveiling an elevated occurrence of this syndrome in sectors characterized by a lower-middle socioeconomic class. This finding aligns with and reinforces previously established evidence. Ultimately, the existing body of evidence, coupled with our study findings, underscores the significance of investigating frailty and its associations with the urban environment and related factors. This emerging field of research holds groundbreaking potential, offering substantial implications for geriatric medicine. Furthermore, it provides invaluable insights that can inform the development of governmental initiatives aimed at promoting healthy aging and proactively preventing frailty.

## Abbreviations

ANOVA: analysis of variance
CEC: Comité de Ética Científica
GIS: geographic information systems
IDE: Infraestructura de Datos Geospaciales
